# Three-Dimensionally Printed, On-Demand, Patient-Specific Breast Models to Support Mastectomy Gross Examination: A Proof-of-Concept Study

**DOI:** 10.7759/cureus.111092

**Published:** 2026-06-18

**Authors:** Panagiotis Kousidis, Antonia Syrnioti, Foteini Pavlidou, Miltiadis Pliachas, Anastasia Nikolaidou

**Affiliations:** 1 Pathology Department, "Theageneio" Anticancer Hospital of Thessaloniki, Thessaloniki, GRC; 2 Breast Surgical Oncology Department, "Theageneio" Anticancer Hospital of Thessaloniki, Thessaloniki, GRC

**Keywords:** 3d printing, breast cancer, gross examination, mastectomy, pathology workflow, patient-specific, radiologic-pathologic correlation, surgical pathology, three-dimensional printing, tumor localization

## Abstract

Accurate gross examination of mastectomy specimens relies on accurate correlation between preoperative imaging and specimen anatomy to guide sectioning, orientation, and margin-oriented sampling. Radiologic-pathologic spatial discordance remains a common source of variability, particularly in multifocal tumors and in neoadjuvant-treated specimens with poorly defined tumor beds. Three-dimensional (3D) printing enables the production of patient-specific physical models that may assist in tumor localization and spatial understanding during macroscopic assessment. In this proof-of-concept study, two representative breast magnetic resonance imaging (MRI) examinations were selected from the DUKE-BREAST-CANCER-MRI dataset of The Cancer Imaging Archive (TCIA). The two cases represented unifocal and multifocal breast cancer, respectively. Segmentation of the breast contour and lesions identified as tumors was performed in both cases, with additional segmentation of a possible enlarged axillary lymph node in the second case. Anatomical orientation labels, a nipple landmark, and empty rectangular labels for each case number were incorporated into both models. The projection of the lesions onto the anterior external breast contour was encoded using protruding lines to indicate their relative surface extent. Each model was printed in separate parts, sectioned along the sagittal plane, to indicate the relative position of the tumor(s) in sagittal cross-section. All models were printed using polylactic acid (PLA) filament on a Bambu Lab A1 3D printer (Bambu Lab, Shenzhen, People’s Republic of China). Printing time and material use were recorded at the maximal print speed preset of 166%. A Pareto analysis, on printer-estimated total print time and slicer-estimated total material use, was performed on the two-tumor model, with identification of the respective Pareto knees. An additional third model was produced from an MRI examination showing a lesion with associated breast skin thickening and enhancement. This model was printed in two colors to aid the identification of the extent of breast skin with MRI abnormalities. For all three models, a cost estimation was performed, incorporating material use, electricity consumption, and final print weight after support removal for the estimation of incineration cost.

In conclusion, MRI-derived, patient-specific 3D-printed breast models may be produced in modest time and at low cost within a pathology-oriented workflow. They may be used not only for spatial orientation but also for educational and research purposes. This proof-of-concept study supports further evaluation of such models as spatial orientation aids for gross sectioning, tumor bed localization, and margin-oriented sampling, particularly in cases with radiologic-pathologic discordance and in neoadjuvant-treated specimens. Future clinical decision support applications would require the development of a validated workflow for model production.

## Introduction

The gross examination of mastectomy specimens for breast cancer can be challenging. The accurate identification and sampling of all lesions may be particularly difficult in the setting of neoadjuvant therapy and poorly defined tumor beds [1]. Multicentric disease may represent another significant challenge in gross examination. The review of clinical history and radiologic findings prior to gross examination, by the pathologist or grossing personnel, is strongly recommended in the literature [2]. However, correlating two-dimensional (2D) images and examination results in text with the three-dimensional (3D) anatomy of a mastectomy specimen may not be straightforward in all cases. The integration of patient-specific, 3D-printed breast models for real-time review at the grossing bench during sectioning may potentially aid in spatial orientation and lesion localization. The low-cost, on-demand, in-house production may reduce both cost and time barriers. We present a workflow for the production of 3D-printed educational models to assist in 3D visualization during the gross examination of mastectomy specimens in the setting of breast cancer.

A prior version of this study has been accepted as a provisional e-poster for presentation at the 38th European Congress of Pathology 2026, to be held in Stockholm, Sweden, from September 12 to 16, 2026.

## Technical report

**Figure 1:**
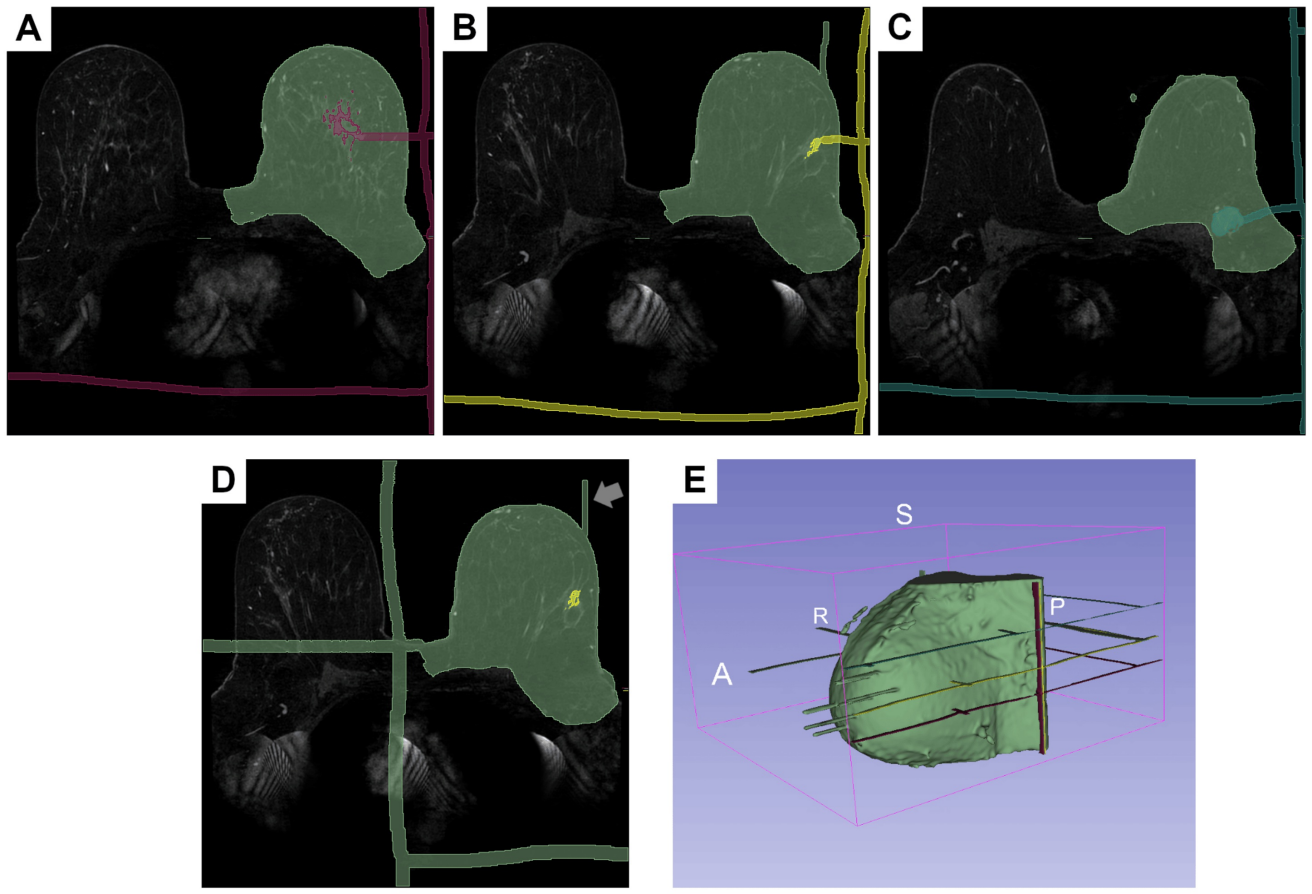
Photographs from the segmentation of the two-tumor model in 3D Slicer In Figure 1A, the first lesion identified as a tumor is masked in magenta color, with the protruding lines toward the borders of the x- and y-orientation axes visible. A similar line exists for the z-axis but is not visible in the current view. These lines were used subsequently for segmentation alignment in Bambu Studio. Similarly, in Figure 1B, the second lesion identified as a tumor is masked in yellow, while in Figure 1C, the possible enlarged axillary lymph node is masked in blue. In Figure 1D, the breast contour is masked in green, with the protruding lines for alignment visible, as well as a line indicating the lateral edge projection of the second lesion identified as a tumor on the breast surface (gray arrow). Finally, in Figure 1E, the 3D-rendered model is shown.

Two representative breast magnetic resonance imaging (MRI) examinations were selected from the DUKE-BREAST-CANCER-MRI dataset of The Cancer Imaging Archive (TCIA) [3]. The Digital Imaging and Communications in Medicine (DICOM) images were imported into the computer software 3D Slicer version 5.8.1. Segmentations of the breast contour, the lesions identified as tumor(s), and a possible enlarged axillary lymph node in the second examination were performed. The breast contour was segmented up to the visible posterior boundary corresponding to the pectoralis fascia. To support relative alignment, protruding lines were designed from each segmented structure to both borders of each of the three orientation axes. Additional protruding lines were designed on the anterior surface of the breast model, corresponding to the projection of the lesion(s), to indicate their surface extent. Photographs from the segmentation process are shown in Figure 1.

**Figure 2:**
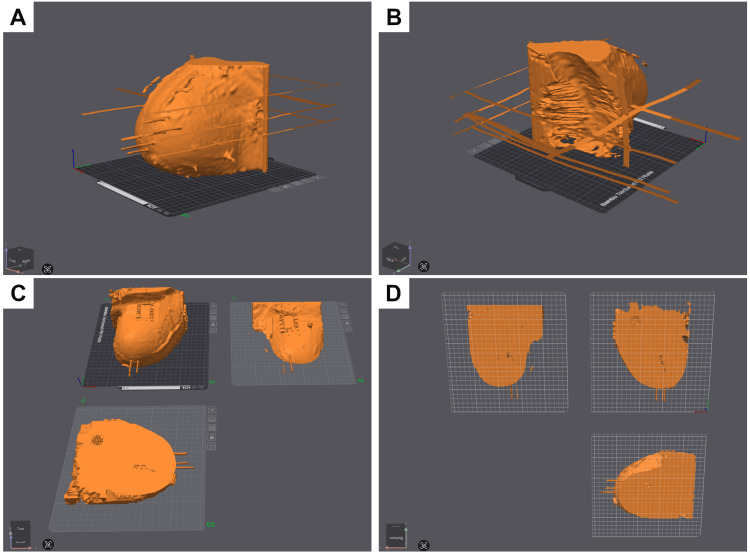
Screenshots from the Bambu Studio slicing process for the two-tumor breast model In Figure 2A, the model is shown in the Bambu Studio environment, in an orientation similar to the rendering shown in Figure 1E. In Figure 2B, the posterior surface of the model is visible, including the imprint corresponding to the pectoralis fascia. In Figure 2C and Figure 2D, the final three parts produced after sagittal sectioning of the model in Bambu Studio are shown, automatically arranged for printing on the print plates.

The segmentations for each representative case were subsequently exported as .stl files and imported into the software Bambu Studio version 2.6.0.51 (Bambu Lab, Shenzhen, People’s Republic of China). For each model, the segmentations were aligned and merged into an assembly. Each segmentation underwent the “Fix model” procedure. The “Mesh Boolean” tool [4] was used to generate each case model by subtracting the segmented lesion volume(s) and the possible enlarged axillary lymph node in the second case from the corresponding breast contour model. Breast orientation labels, a nipple landmark, placeholder labels for pseudonymized case numbers, and a marking grid for the possible enlarged lymph node cavity were designed on the software Autodesk TinkerCAD (Autodesk Inc., San Francisco, CA), imported into Bambu Studio, added to each case model, merged, and positioned appropriately. Photographs from the Bambu Studio processing of the models are shown in Figure 2.

Printing was performed using a Bambu A1 3D printer, a 0.4 mm hardened steel hotend, and a Bambu Textured Polyetherimide (PEI) plate. The printing technique used was fused deposition modeling (FDM). The filament used was eSUN PLA+ 1.75 mm Orange (Shenzhen eSUN Industrial Co., Ltd., Shenzhen, People’s Republic of China). The Bambu Automated Material System (AMS) Lite was connected to the printer for filament feeding. The system preset of “0.20mm Standard @BBL A1” was used, with the modifications of “Wall loops” set to 1, “Sparse infill density” set to 3%, and “Enable support” selected. In the “send print job” dialog box, the settings of “Flow Dynamics Calibration” and “Auto Bed Leveling” were enabled for each print.

**Figure 3:**
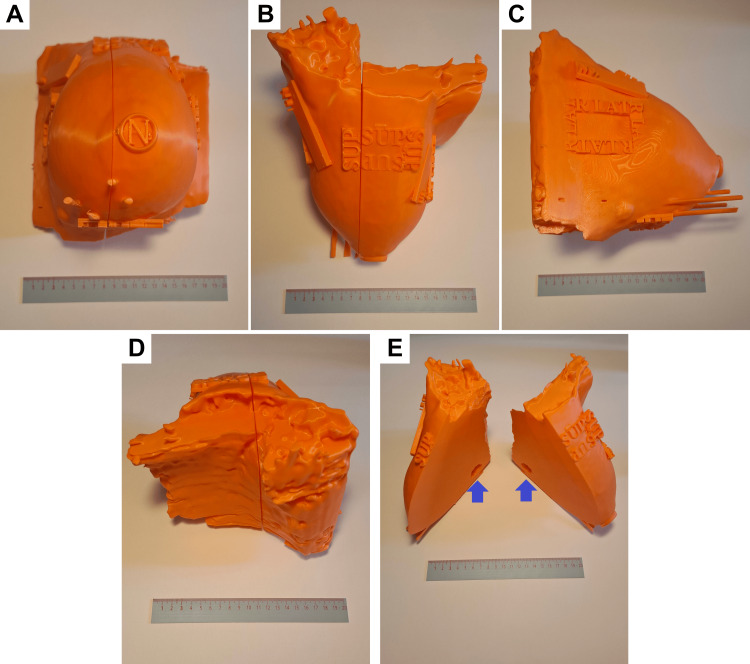
Photographs of the printed single-tumor model The model was printed in two parts, sectioned along the sagittal plane at the level of the lesion identified as a tumor. Figure 3A shows the anterior view, Figure 3B the superior view, and Figure 3C the right lateral view. In Figure 3D, the posterior surface of the model is visible, including the imprint corresponding to the pectoralis fascia. In Figure 3E, the two printed parts are shown with their inner surfaces visible. The parts of the cavity corresponding to the lesion identified as a tumor are indicated by the blue arrows.

**Figure 4:**
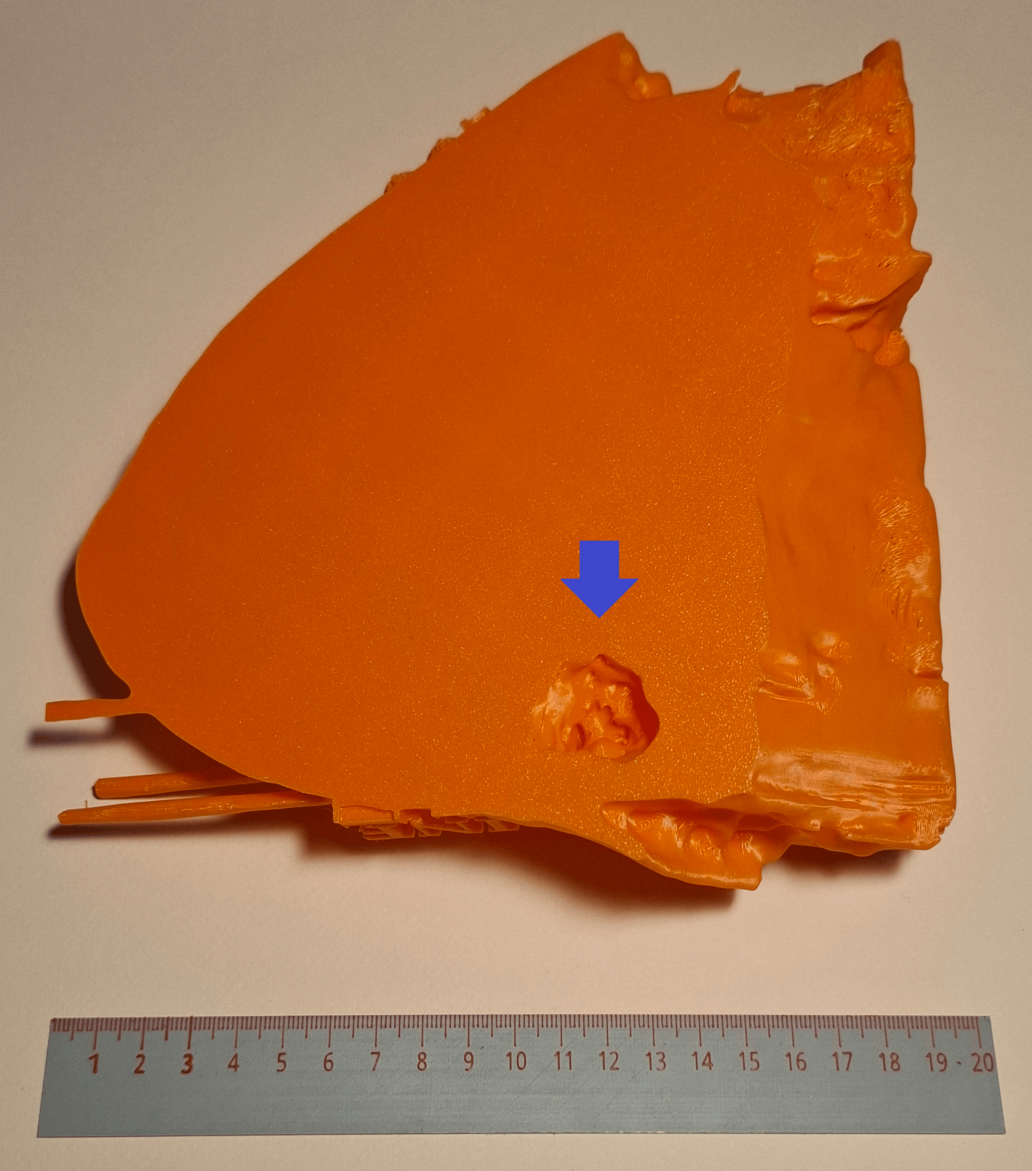
Photograph from the printed sagittal section of the single-tumor model In this view, the cavity corresponding to the lesion identified as a tumor is visible (blue arrow).

**Figure 5:**
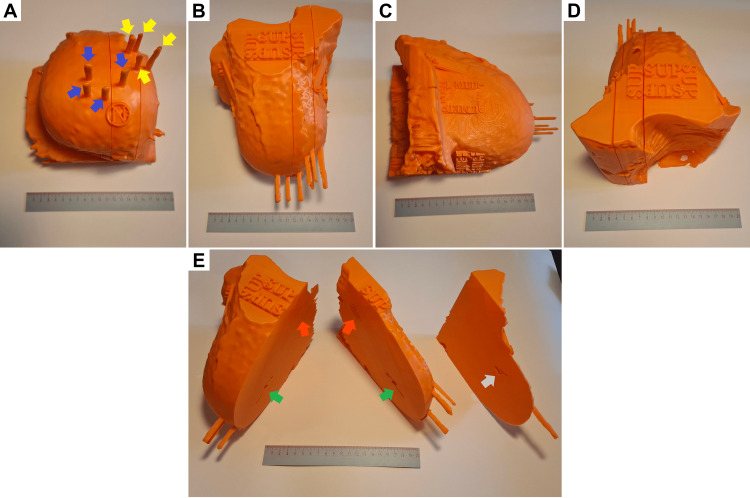
Photographs from the printed two-tumor breast model The model was printed in three parts, sectioned along the sagittal plane, to highlight the two lesions identified as tumors and the possible enlarged axillary lymph node. Figure 5A shows the anterior view. The protrusions indicating the projections of the two lesions onto the breast surface are marked by the blue and yellow arrows, respectively. Figure 5B shows the superior view, while Figure 5C shows the right lateral view. In Figure 5D, the posterior surface of the model is visible, including the imprint corresponding to the pectoralis fascia. Figure 5E shows the three printed parts with their inner surfaces visible. The green and gray arrows indicate the first and second lesions identified as tumors, respectively, while the red arrows indicate the cavity corresponding to the possible enlarged axillary lymph node, which is covered with a grid design to reduce potential confusion during gross examination.

**Figure 6:**
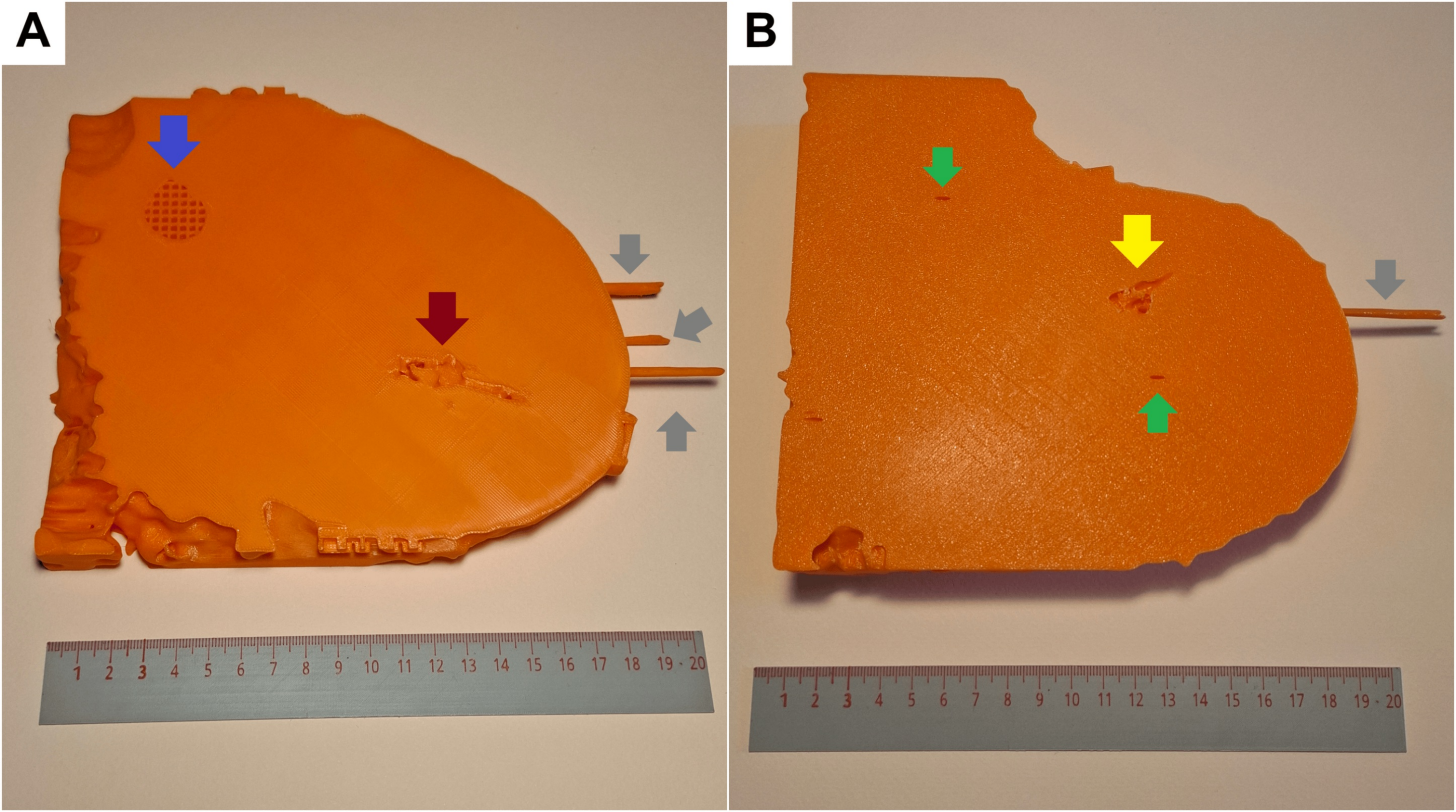
Photographs from the printed sagittal section parts of the two-tumor model In Figure 6A, the red arrow indicates the first, larger lesion identified as a tumor. The blue arrow indicates the position of the possible enlarged axillary lymph node. The cavity created by the subtraction of the lymph node segmentation from the breast parenchyma was covered with a grid to indicate that it represents a separate structure and to reduce potential confusion during gross examination. The gray arrows indicate projections of the borders of the first lesion onto the anterior breast surface. In Figure 6B, the yellow arrow indicates the second lesion identified as a tumor. The green arrows indicate small voids created by the Mesh Boolean subtraction of the alignment markings; these are technical artifacts and have no intended anatomical significance. The gray arrow similarly indicates a projection of the border of the second lesion onto the anterior breast surface.

Each model was printed in multiple parts, as sections along the sagittal plane at the lesion position(s), performed with the “Cut” tool in Bambu Studio. The models were sliced in Bambu Studio, exported as plate-sliced files for each print plate in .gcode.3mf format, and transferred locally to the printer via Secure Digital (SD) card, without cloud-based transmission. Printing was performed at 100% model scale using the maximum 166% print speed preset. After print completion, the print supports were manually removed. Photographs of the single-tumor model are shown in Figure 3 and Figure 4, and a corresponding video is available as Video 1. Photographs of the two-tumor model are shown in Figure 5 and Figure 6, and a corresponding video is available as Video 2.

**Video 1:** Showcase of the single-tumor, two-part, 100% scale model The model is represented in an assembled form, with the two separate parts connected with adhesive tape.

**Video 2:** Showcase of the two-tumor, three-part, 100% scale model The model is represented in an assembled form, with the three separate parts connected with adhesive tape.

The print time and final measured print weight were recorded. The final measured print weight was calculated as the sum of filament purge, weight of removed supports, and weight of the final model parts.

**Figure 7:**
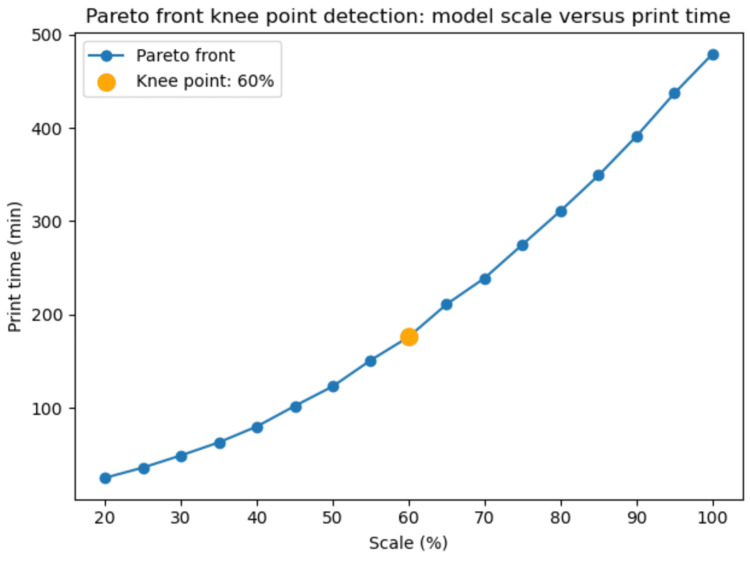
Pareto-knee analysis of total printer-estimated print time versus model scale at the 166% print speed preset The plot shows total printer-estimated print time, in minutes, across model scales from 20% to 100%. The Pareto knee was identified at 60% scale, corresponding to a total printer-estimated print time of 176 minutes.

**Figure 8:**
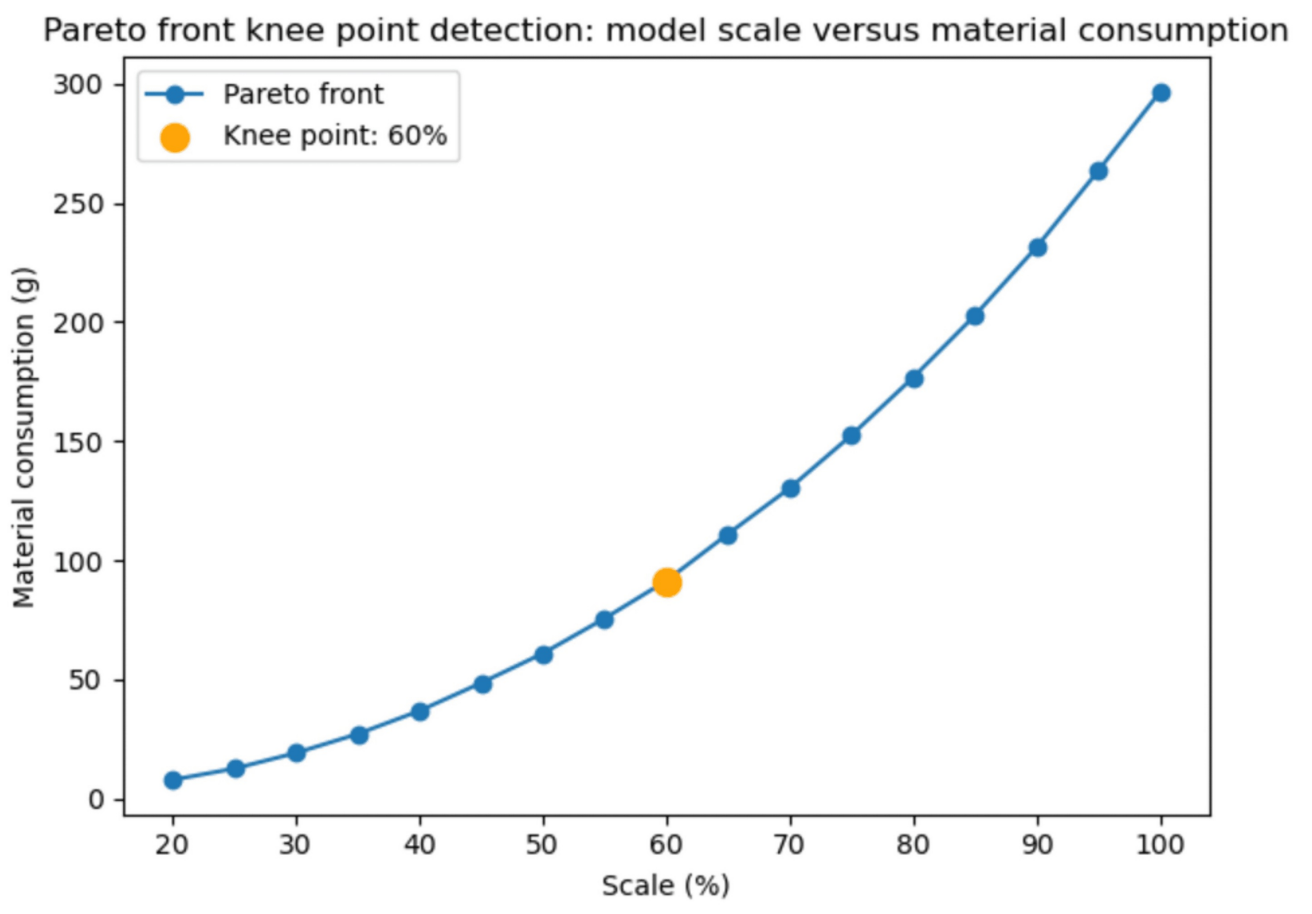
Pareto-knee analysis of total slicer-estimated material use versus model scale The plot shows total slicer-estimated material use, in grams, across model scales from 20% to 100%. The Pareto knee was identified at 60% model scale, corresponding to a total slicer-estimated material use of 91.37 g.

In order to identify a pragmatic compromise for large-scale 3D printing, two separate Pareto analyses were performed for the two-tumor model, evaluating the trade-off between anatomic scale preservation and reductions in print time and material consumption, respectively [5]. The analyses separately evaluated the total printer-estimated print time and total slicer-estimated material consumption against model scale. Initially, the three parts of the two-tumor model were scaled down in consecutive 5% increments, from 100% down to 20% scale, and rearranged on the print plates with the “Arrange all objects” tool, with “Auto rotate for arrangement” enabled, in Bambu Studio. Subsequently, the print files were sent to the 3D printer via local area network (LAN) connection, with the use of the “LAN only mode” of the Bambu A1 printer, without cloud-based transmission. Each plate was sent to the printer, and the 166% print speed preset was selected. The printer-estimated print time and the slicer-estimated material use for each plate were recorded. The total printer-estimated print time and slicer-estimated material use for each print scale were calculated as the sum of all respective plates for a given model and print scale. Pareto analyses were performed in Anaconda JupyterLab version 4.4.7 using Python 3 and the numpy, matplotlib, paretoset, and kneed packages. With the predefined objective of maximizing printing scale while minimizing print time and material consumption, all recorded scale options corresponded to Pareto-optimal solutions in both analyses. Knee-point detection was then performed using the kneed package, which implements the Kneedle algorithm [6], to identify a pragmatic compromise point across the resulting Pareto front. The Pareto plots for the two-tumor model, with a highlight of the Pareto front knee point, on printer-estimated total print time and slicer-estimated total material use against model scale, are included in Figure 7 and Figure 8, respectively.

To quantify additional cost dimensions, the electricity consumption for each 3D print was measured using a Tapo P110 (TP-Link Systems Inc., Irvine, CA), connected between the 3D printer plug and the wall outlet. The measurement was performed from the onset of printing, with the hotend and printer bed at room temperature, until print completion and return of the nozzle temperature to 50°C.

**Figure 9:**
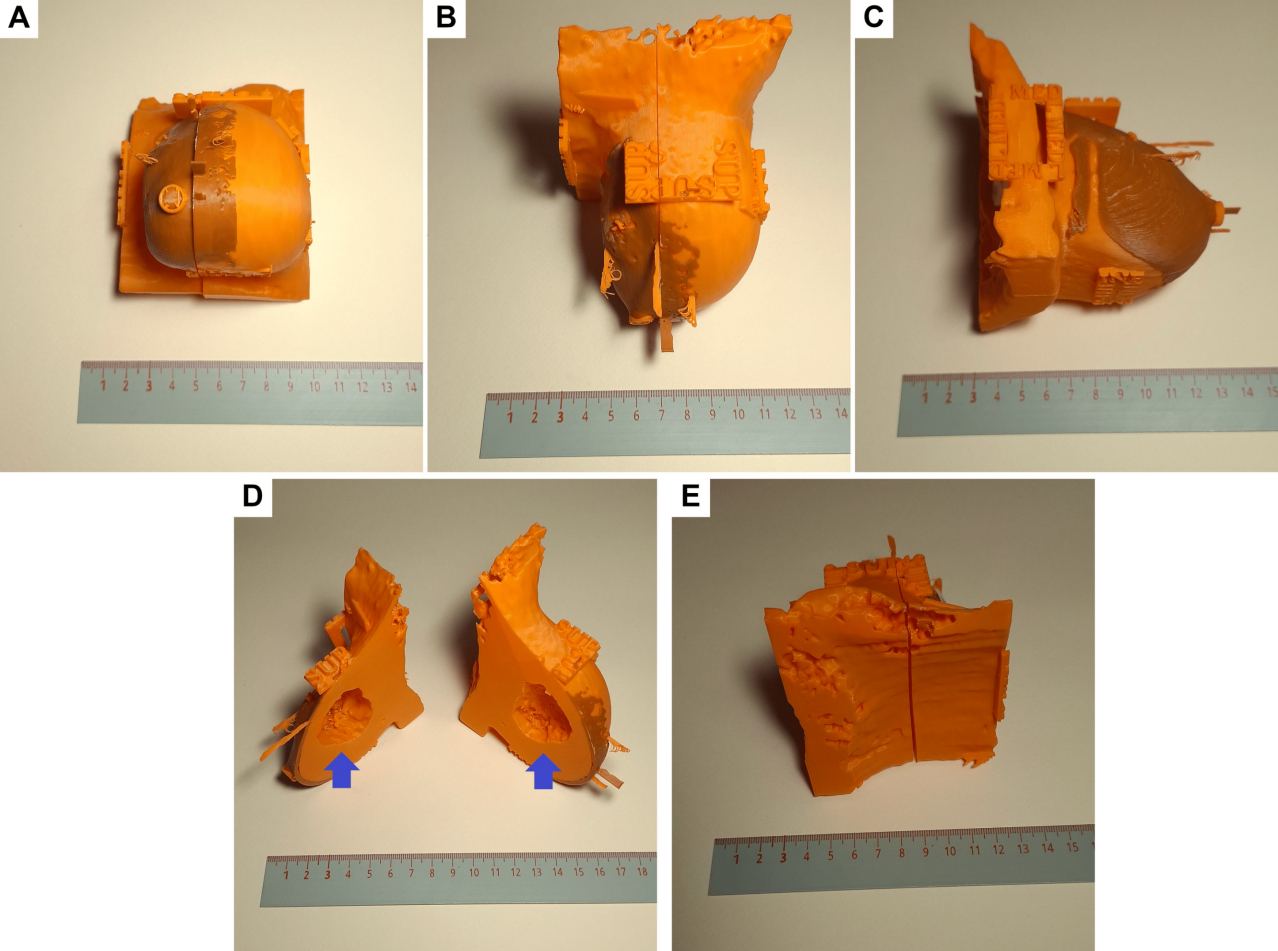
Photographs of the two-color, single-tumor breast model showing the area of skin thickening identified on MRI The area corresponding to skin thickening is printed in brown. Figure 9A shows the anterior view, Figure 9B the superior view, and Figure 9C the right lateral view. In Figure 9D, the two printed parts are shown with their inner surfaces visible. The cavity corresponding to the lesion identified as a tumor is indicated by the blue arrows. In Figure 9E, the posterior surface of the model is visible, including the imprint corresponding to the pectoralis fascia. MRI: magnetic resonance imaging

A third representative MRI examination, showing a lesion identified as a tumor together with associated breast skin thickening and enhancement, was also selected. The same methodology was followed, with additional separate segmentation of the skin thickening and enhancement area. The lesion identified as a tumor was subtracted from the breast model using the “Mesh Boolean” tool with the subtraction method. The segmented area of skin thickening was resized and positioned on the surface of the final model to indicate the extent of the imaging abnormality. The model was printed in two parts, as sections along the sagittal plane, and in two colors using the Bambu AMS Lite. The filament used for the area of skin thickening was Creality Hyper PLA RFID 1.75 mm Brown (Shenzhen Creality 3D Technology Co., Ltd., Shenzhen, People’s Republic of China). Printing was performed at 50% scale. Photographs of this third model are shown in Figure 9, and a corresponding video is available as Video 3.

**Video 3:** Showcase of the two-color, single-tumor, two-part, 50% scale model The model is represented in an assembled form, with the two separate parts connected with adhesive tape.

An estimation of the cost of incineration was performed, with measurement of the final model weight after support removal. All weight measurements were performed with a digital scale of 1 g resolution. For each weight measurement, the digital scale was zeroed before use, and 10 consecutive measurements were obtained. The mean value was recorded for analysis.

Total segmentation and model preparation time, print time, material consumption and estimated total material cost, electricity consumption and estimated electricity cost, and estimated cost of incineration are included in Table 1.

**Table 1:** Print time, material consumption, electricity consumption, and estimated cost of the 3D-printed breast models Electricity cost was estimated using a local electricity cost of 0.12 EUR/kWh [7]. Incineration cost was estimated using a cost of 1.70 EUR/kg of final measured model weight after support removal [8]. The total estimated cost was calculated as the sum of the estimated total material cost, estimated electricity cost, and estimated incineration cost. The prices of 24.99 EUR per 1 kg spool of eSUN PLA+ and 34.99 EUR per 1 kg spool of Creality Hyper PLA RFID were used to calculate the estimated total material cost. Prices reflected vendor-listed EU store prices valid as of June 12, 2026; sale discounts were not applied. In the two-colored model, the relative material use for cost estimation was calculated by the weight difference of the respective spools before and after printing. Cost estimates were rounded to two decimal places.

Model	Total segmentation and model preparation time	Total print time at 166% print speed preset	Measured weight of filament purge and supports (g)	Final measured model weight after support removal (g)	Total measured electricity consumption (kWh)	Estimated total material cost (EUR)	Estimated electricity cost (EUR)	Estimated incineration cost (EUR)	Total estimated cost (EUR)
Single-tumor, two-part, 100% scale	49 m 31 s	6 h 24 m	16.7	204.3	0.665	5.52	0.08	0.35	5.95
Two-tumor, three-part, 100% scale	1 h 1 m 8 s	7 h 55 m	19	265.8	0.967	7.12	0.12	0.45	7.69
Two-color, single-tumor, two-part, 50% scale	1 h 7 m 36 s	4 h 18 m	49.3	50.9	0.581	2.71	0.07	0.09	2.87

## Discussion

The gross examination of mastectomy specimens can be particularly challenging. Multicentric disease and/or administration of neoadjuvant treatment may increase the difficulty of accurate lesion identification and sampling. Specimen radiography, after potential prior placement of radiopaque clips, may be employed to aid in lesion localization [1,9]. Radiography remains of particular importance in the setting of microcalcifications, the identification of which, as they are not visible on MRI [10], may not be facilitated by 3D-printed models based on MRI examinations. Conversely, radiopaque clip migration during placement, as well as clip loss during surgery or subsequent pathologic manipulation of the specimen, has been documented in the literature [11]. Because the present workflow uses MRI examinations rather than radiographic guidance for model construction, such occurrences would not be expected to substantially affect model accuracy.

The prior review of the patient record and all examinations performed, including radiologic examinations and results of previous biopsies, is generally considered a prerequisite for mastectomy gross examination [1,2,12]. However, translating information from text-based reports, printed 2D images, or even imaging examinations reviewed on a computer screen, to the 3D anatomy of a mastectomy specimen, may be suboptimal. Real-time consultation of patient-specific 3D-printed models has already been described in other disciplines and has been associated with positive outcomes. In a prospective clinical study of 61 patients undergoing right hemicolon surgery, Chen et al. evaluated the use of 3D printing or 3D imaging to visualize the superior mesenteric vessels for the surgical team. The 3D printing group was associated with statistically significant reductions in duration of surgery, intraoperative bleeding volume, and medical expenses, in comparison to the control group. The authors also highlighted the practical value of real-time model consultation, with the model positioned near the laparoscopic screen for intraoperative reference [13]. The placement of a 3D-printed model next to the grossing bench for real-time consultation during mastectomy gross examination represents a conceptually similar approach. In pathology, the absence of a sterile operative field in most grossing settings allows the model to be handled directly during specimen examination, which may facilitate repeated spatial reference during sectioning and sampling.

The optimal print scale of the models can be debated. It could be suggested that, for mastectomy specimens sampled fresh, before formalin fixation, a 100% scale model may provide the closest dimensional comparison. Regarding the effects of formalin fixation on a breast excision specimen, although the literature harbors mixed results, the relevant studies predominantly report a relatively stable tumor size with shrinkage of adjacent tissues [14,15], with a potential to result in a lower estimated distance to margins. This expected reduction in breast size from fixation-related changes may be considered to print the model in a lower scale, in order to produce a final model with comparable dimensions. However, the aforementioned literature results indicating differential shrinkage may complicate this approach, as the downscaling of the entire 3D model could create a mismatch between lesion size and surrounding breast tissue. The 3D-printed models may be more applicable in the fresh grossing setting, where relative breast tissue and lesion volumes and positions have not been altered.

In the 3D printing of patient-specific models, a reduction in scale of 50% has been proposed in order to reduce print cost [16]. The potential selection of a reduced printing scale may be more complex in cases with smaller breast volumes and smaller tumors, as downscaling may further reduce the visibility of clinically relevant structures. Further studies are required to define the most appropriate scaling strategy for pathology-oriented breast models in this setting.

Pareto analysis can be used for parameter optimization by identifying an efficient trade-off between competing variables. This may be applicable as a cost and time optimization strategy in large-scale model production, as an alternative to default production at 100% scale. In Figure 7 and Figure 8, the respective Pareto-knee points are highlighted in orange. The knee points indicate the scale at which further downscaling is associated with diminishing reductions in print time or material consumption. In the present analysis, both total printer-estimated print time and slicer-estimated material consumption showed a knee point at 60% model scale. This finding suggests that approximately 60% scale may represent an efficient compromise between retained model size and production-resource requirements for this specific model. Further research is required to determine the most appropriate optimization strategy for the production of breast models in this setting.

The material PLA was selected for this study because it is a widely used, cost-effective FDM material with favorable printability. Previous studies have reported the use of 3D-printed PLA in biomedical and clinical settings, including prostheses, surgical instruments, medical education models, and simulators [17].

Printing the models in separate parts may help highlight the relative position of the lesion(s), while sagittal sectioning was selected because it reflects the conventional sectioning plane used in current practice [2]. It should be noted that, in addition to an increased model scale, printing models in multiple parts due to multiple lesions may increase print time, particularly when the model parts cannot fit on a single build plate and multiple separate prints are required. Similarly, printing in multiple colors requires automatic filament changes through the AMS Lite and is generally also expected to increase print time. In our study, the two-tumor, three-part model at 100% scale required the longest print time, whereas the two-color, single-tumor, two-part model at 50% scale required less time, consistent with the combined influence of model scale, number of printed parts, and utilization of multicolor printing.

The proposed workflow has several advantages. The models were produced using a commercially available desktop 3D printer and widely accessible software, limiting direct production costs to the initial purchase of the printer, the filament, the electricity, the operator time, and the printer maintenance. The final models produced in this proof-of-concept study are early indications that overall production costs may be low, while they may be reduced even further when the models are printed at a reduced scale. Multicolor printing may also be incorporated to highlight additional areas of interest, such as breast skin with increased MRI signal. The workflow also has several limitations. Firstly, this strategy is applicable to solid tumors and mass lesions and is not suitable for areas of microcalcifications. Secondly, 3D printing has safety considerations and requires adherence to best practices and manufacturer instructions for use (IFUs). Thirdly, the current workflow may be utilized only for the production of educational and spatial orientation aid models, to assist in the 3D visualization and relative position of tumors in mastectomy gross examination. If the initial segmentations are accurate, it can be expected that lesion dimensions, distances to the skin, nipple, and thoracic wall, and relative distances between multiple lesions can be measured and will be proportionate with scaled models. However, the models should not currently be used for this purpose in clinical decision-making, as this would constitute a clinical decision support application. Such use would require a validated segmentation pipeline, approved software and hardware, documented accuracy, evidence of clinical benefit, traceability, and quality management procedures. This represents a promising future research direction. A head-to-head comparison of various combinations of radiologic examination review, patient-specific 3D-printed breast models, and specimen radiography would provide a more robust evaluation and help quantify any potential benefit, particularly in settings where specimen radiography may not be available. Incorporating specimen dimensional changes after fixation could allow these models to be adapted for use with fixed mastectomy specimens.

The production of the models requires access to breast MRI examinations. Collaboration with experts in radiology is necessary to ensure accurate segmentation and the subsequent generation of accurate final models. This process, however, may be time-consuming, especially if performed serially for the production of multiple models for different cases. In our study, because segmentations were performed manually by an operator without specialized radiologic segmentation training, greater proficiency with 3D Slicer may reduce segmentation time.

Patient-specific, 3D-printed breast models have previously been described in the literature. A feasibility study was published in 2021 by Santiago et al., studying the decrease in decisional conflict in patients with breast cancer, when patient-specific 3D models are utilized in physician-patient discussions [18]. This application differs from the present workflow, which is intended for grossing personnel and not optimized for patient communication. Potentially, a combined workflow could be constructed, in which the same initial segmentations are utilized for the production of two separate types of models for the same patient. One model could be used for physician-patient communication, while the other model could support gross examination in pathology. This is a further promising research direction. Additionally, 3D printing has been utilized for surgical guides for breast-conserving surgery, surgical planning, and intradisciplinary communication [19-21]. However, the use of patient-specific 3D-printed models to guide gross examination in pathology represents a novel application of this technology.

The environmental aspect of the 3D-printed models should also be considered. As PLA is compostable in specific conditions, but relatively stable in conventional landfill environments, a collaboration with an appropriate composting facility could potentially mitigate the environmental impact [22,23]. An additional consideration is contamination with biological material if the models are handled at the grossing bench simultaneously with specimen gross examination. In such cases, disposal according to local biohazardous waste procedures, including sterilization or incineration where required, may be necessary. This would produce a small additional cost [8].

## Conclusions

The on-demand production of patient-specific breast models to support pathology gross examination appears technically feasible using the described workflow and may be associated with low production cost. In the current proof-of-concept setting, these models should be regarded as educational and spatial-orientation aids rather than validated clinical decision support tools. Further research is required to determine whether their integration into pathology workflows improves gross examination time, lesion localization, sampling strategy, number of sections submitted, specimen revisits, radiologic-pathologic correlation, or turnaround time. The future development of a validated workflow for the segmentation and production of patient-specific 3D-printed breast models, and the confirmation of the model accuracy and benefits in clinical implementation, would be necessary before such models could be used to support clinical decision-making during mastectomy gross examination.
